# Appearance of Focal Nodular Hyperplasia after Chemotherapy in Two Patients during Follow-Up of Colon Carcinoma

**DOI:** 10.1155/2021/6676109

**Published:** 2021-04-03

**Authors:** L. A. de Wert, S. A. Huisman, F. Imani, D. J. de Gooyer, J. M. G. H. van Riel, P. D. Gobardhan, A. M. Rijken

**Affiliations:** ^1^Department of Surgery, Amphia Hospital, Breda, Netherlands; ^2^Department of Radiology, Amphia Hospital, Breda, Netherlands; ^3^Department of Oncology, Bravis Hospital, Roosendaal/Bergen op Zoom, Netherlands; ^4^Department of Oncology, ETZ Elisabeth Hospital, Tilburg, Netherlands

## Abstract

Surgical liver resection is a treatment option in patients with resectable colorectal liver metastases. We present two cases of focal nodular hyperplasia (FNH) development after treatment with oxaliplatin during follow-up of colon carcinoma. The first case was a 40-year-old male patient who developed multiple liver lesions suspect for metastatic disease four years after he had undergone laparoscopic right-sided hemicolectomy and adjuvant chemotherapy (capecitabine and oxaliplatin). He underwent a metastasectomy of segments three and four and microwave ablation (MWA) of the lesion in segment one. Pathological analysis demonstrated FNH. The second patient was a 21-year-old woman who presented with multiple liver lesions during follow-up for colon carcinoma. She underwent a laparoscopic right-sided hemicolectomy and was adjuvantly treated with capecitabine and oxaliplatin three years ago. Magnetic resonance imaging (MRI) was performed, and the lesions showed no signs of metastatic disease but were classified as FNH. Therefore, the decision was made to follow up the patient. In conclusion, the development of benign liver lesions could occur during follow-up of colon carcinoma and might be caused by oxaliplatin-induced changes to the liver parenchyma. Hence, it is important to distinguish these from metastatic liver disease.

## 1. Introduction

Colon carcinoma is one of the most common cancers in the world. About 18-25% of patients are diagnosed with liver metastasis in less than five years of the primary diagnosis [1, 2]. In order to prevent future metastatic disease in patients with surgically resected high-risk stage 2 or stage 3 colon carcinoma, adjuvant treatment with oxaliplatin-based chemotherapy is the standard of care [3]. In addition, routine abdominal imaging is performed during follow-up to early detect metastatic liver disease, because surgical treatment of liver metastasis improves survival rates drastically [4]. Although improving, surgical treatment is still associated with high morbidity rates [5], so it is important to critically analyze patients with a hepatic mass suspect for metastatic liver disease. Here, we present two cases of the development of FNH during follow-up of colon carcinoma after adjuvant treatment with oxaliplatin-based chemotherapy.

## 2. Case Presentation

### 2.1. Case 1

**Figure 1:**
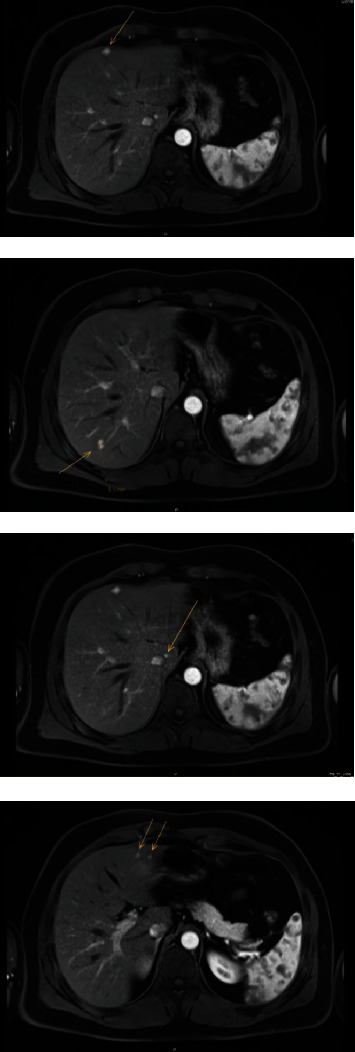
Liver MRI: demonstrates several liver lesions (segments 1, 3, 4, 7). These lesions were new compared to the liver MRI five years ago. One lesion in segment 3 and one lesion in segment 1 were new compared to the MRI scan of one month ago.

**Figure 2:**
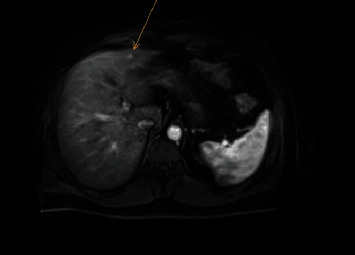
Liver MRI one month earlier demonstrated only one lesion in segment 3 and no lesion in segment 1.

A 40-year-old male patient with a history of pT3N1M0 colon carcinoma, who had undergone laparoscopic right-sided hemicolectomy four years ago (2015) and was subsequently treated with eight courses of capecitabine and oxaliplatin regimen as adjuvant chemotherapy, was referred to our hospital's specialised liver surgery unit because of multiple focal liver lesions suspicious for metastatic disease. During routine follow-up in 2019, two small liver lesions were discovered on ultrasound. At that time, the concentration of carcinoembryonic antigen (CEA) was low (1.7 *μ*g/L). Subsequently, contrast-enhanced MRI of the liver was performed which showed seven lesions suspect for metastatic disease (Figure 1). In addition, two lesions were new compared to the MRI performed one month earlier in another hospital (Figure 2). Because of the high suspicion of metastatic disease, a decision was made to treat the liver lesions. The patient underwent a metastasectomy of segments three and four and MWA of the lesion in segment one.

Surprisingly, pathological analysis did not reveal signs of malignancy or metastatic disease in the resected specimen. Instead, the lesions consisted of fibrous septa with a central stellate scar which is characteristic for focal nodular hyperplasia (FNH). Postoperative MRI after three months did not demonstrate any new lesions.

### 2.2. Case 2

**Figure 3:**
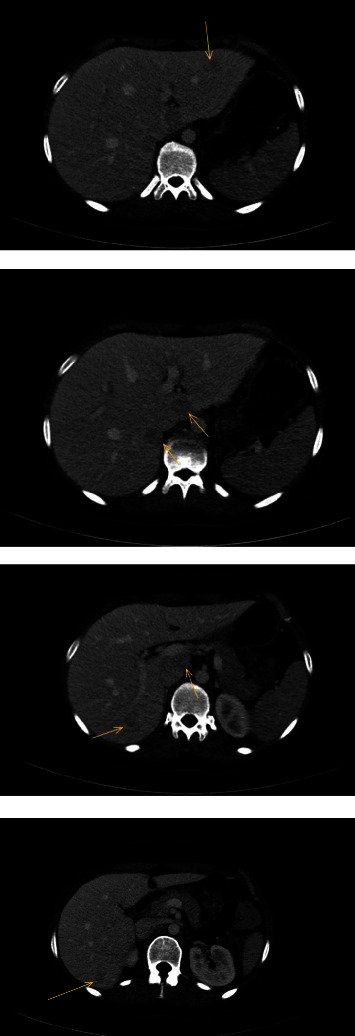
Liver CT demonstrated the onset of seven liver lesions suspect for metastatic disease.

**Figure 4:**
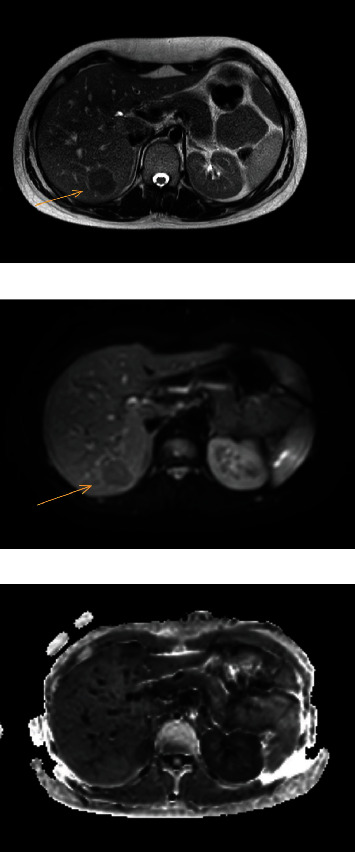
The lesions were isointense in T1-weighted images and hyperintense in T2-weighted images and did not demonstrate any diffusion restriction.

A 21-year-old woman with Lynch syndrome and a history of pT3N1M0 colon carcinoma underwent a laparoscopic right-sided hemicolectomy and received eight courses of adjuvant capecitabine and oxaliplatin. During follow-up three years after her initial treatment, she presented with weight loss but without other complaints. The CEA level was low (<1.0 *μ*g/L), but a thoracoabdominal CT scan demonstrated multiple focal liver lesions suspicious for metastatic disease (Figure 3). Liver MRI with and without gadoxetate sodium was performed (Figure 4). The lesions were hyperintense in the arterial and venous phase and hypointense in the late arterial phase (central scar). They did not show any diffusion restriction. In addition, a positron emission tomography (PET) scan did not reveal any uptake or signs of metastases. Therefore, the lesions were characterized as FNH and the decision was to reevaluate the patient after three months. Liver MRI three months later did not show any new lesions or an increase in lesions.

## 3. Discussion

These cases illustrate the development of multiple FNH lesions during follow-up after colon surgery combined with adjuvant chemotherapy for colon carcinoma. One patient underwent liver surgery and MWA because of high suspicion of metastatic disease. Pathological analysis, however, demonstrated the presence of FNH. In the other patient, the decision was made to not perform liver surgery, because the lesions were radiological characterized as FNH.

FNH is characterized as a benign liver lesion and represents one of the most common liver neoplasms. It is characterized by hyperplastic hepatocytes and mostly consists of a central stellate scar [6]. The prevalence of FNH in ultrasound studies ranges between 0.8 and 3.2% with multiple lesions in 19% of patients [7]. Its clinical course is mostly asymptomatic, and therefore, FNH is often diagnosed incidentally during routine abdominal imaging. Ultrasound and CT scan are the preferred methods to detect for colorectal liver metastases, but MRI is the modality of choice in diagnosing focal hepatic masses. The sensitivity and specificity of MRI in detecting FNH are about 70-98% [8]. Typically, FNH appears homogenous on MRI imaging with a T1 iso- to hypointense signal and somewhat hyperintense signal on T2-weighted imaging. Sometimes, a hyperintense central scar is seen in T2 or on contrast-enhanced MRI [9]. Colorectal liver metastasis appearance is highly variable on MRI but is mostly hypo- to isointense on T1-weighted images and iso- to hyperintense on T2-weighted images. Peripheral continuous rim enhancement after the addition of gadolinium chelate contrast is a reliable feature of colorectal liver metastasis. Liver biopsy is also an option to differentiate between metastatic and benign liver diseases. However, we try to avoid biopsy for metastatic liver disease in our hospital to prevent the development of biopsy tract metastasis and hematoma [10].

Although the exact aetiology of FNH is unknown, it is thought to be initiated by local changes in liver perfusion causing a hyperplastic regenerative response of the liver parenchyma. It is interesting to note that FNH could be caused by both hyper- and hypoperfusion of the hepatocytes [11].

The development of FNH mimicking metastatic disease after colon surgery with adjuvant chemotherapy is rare. A quick survey under specialised liver surgeons in the Netherlands did not show any other patients who developed FNH after oxaliplatin-based chemotherapy during follow-up after colon surgery. Only a few published case studies describe the development of FNH after colon surgery and adjuvant treatment with oxaliplatin [12–14]. An important side effect of oxaliplatin is the onset of sinusoidal obstruction syndrome (SOS), because it has toxic effects on sinusoidal endothelial cells. SOS decreases liver oxygen saturation [15], increases the expression of hypoxia-induced factors, and stimulates angiogenesis by the upregulation of angiogenic factors [16, 17]. It is hypothesized that SOS and its associated liver hypoperfusion could lead to the formation of benign regenerative lesions such as FNH [3, 18]. A histological study performed by Rubbia-Brandt et al. demonstrates the prevalence of oxaliplatin-induced liver damage in patients who had undergone liver resection [16]. Interestingly, the pathological appearance of SOS and nodular regenerative hyperplasia among these patients (*n* = 247) was 54% and 24.5%, respectively. In contrast, there were no lesions described in the group of patients treated with surgery alone (*n* = 111). Therefore, it must be noted that benign liver alterations can occur after treatment with chemotherapy.

In conclusion, these two cases illustrate the development of FNH during the follow-up of colon carcinoma after treatment with oxaliplatin. Although rare, it is important for physicians to be aware of the onset of benign lesions that mimic metastatic disease possibly caused by the hepatotoxic effect of oxaliplatin in order to prevent unnecessary invasive procedures.

## Data Availability

The data is available on reasonable request.
